# The Relationship between Energy Poverty and Individual Development: Exploring the Serial Mediating Effects of Learning Behavior and Health Condition

**DOI:** 10.3390/ijerph18168888

**Published:** 2021-08-23

**Authors:** Yiming Xiao, Han Wu, Guohua Wang, Shangrui Wang

**Affiliations:** College of Public Administration, Huazhong University of Science and Technology, Wuhan 430074, China; xiaoe@hust.edu.cn (Y.X.); mpawin@hust.edu.cn (G.W.); m202074914@hust.edu.cn (S.W.)

**Keywords:** energy poverty, individual development, health condition, serial mediation

## Abstract

Energy poverty has negative impacts on the residents’ life from various aspects. A comprehensive understanding of these impacts is the top priority in energy poverty governance. Previous qualitative studies have shown that energy poverty has the potential to negatively impact the individual development of residents through multiple pathways. However, few scholars have explored this issue from a quantitative perspective. To fill the gaps in existing research, this study aims to examine the impact of energy poverty on individual development and explore the serial mediating effects of learning behavior and health condition in the relationship. A total of 2289 valid samples are obtained from the dataset of Chinese General Social Survey (CGSS). SPSS 26.0 and PROCESS 3.5 are used to conduct serial mediating effects analysis. The results show that energy poverty can significantly negatively impact the individual development of residents. Learning behavior and health condition are found to independently or serially mediate the relationship between energy poverty and individual development. Health condition has the stronger mediating effect, whereas the mediating effect of learning behavior is weaker. This study may contribute to a better understanding of the consequences of energy poverty in government and academia.

## 1. Introduction

Energy plays an increasingly important role in modern society [1]. Sufficient energy is the prerequisite for residents to live a decent life [2]. However, there are still large numbers of people who have to use primitive solid fuel such as firewood and coal for cooking and heating, or be burdened with heavy debt for purchasing modern energy services [3,4]. Energy poverty, as an emerging concept to define above situation, is gradually attracting the attention of government and academia [5]. It is estimated that many countries in Asia and Africa have more than half of their total population living in energy poverty [6]. During the past decade, energy poverty situations in some countries have even worsened due to lagging governance and economic depression [7].

The concept of energy poverty emerged during the oil crisis in the 1970s [8]. In 1991, Boardman proposed that families were in energy poverty if they had to spend more than 10% of their income to get necessary modern energy services [9]. On the basis of “10% indicator”, many scholars have defined energy poverty in terms of household income and energy consumption, such as Low Income-High Cost index (LIHC), Minimum Income Standards (MIS) and Double Median Expenditure (2M) [10,11,12]. In Warm Homes and Energy Conservation Act 2000, UK government defined energy-poverty people as “members of a household living on a lower income poverty in a home which cannot be kept warm at reasonable cost” [13]. In order to reflect the complexity of the nexus between access to modern energy services and human development, Nussbaumer has underlined the multidimensional nature of energy poverty and proposed the Multidimensional Energy Poverty Index (MEPI) [2]. He has included the adequacy of household modern energy services (cooking, lighting, entertainment/education, etc.) into the identification of energy poverty. According to the study of Villalobos, energy poverty has been defined as a condition of a household experiencing systematic underachievement in energy-related dimensions that could negatively affect functions such as education and health [14]. Furthermore, Villalobos has proposed Perception-based Multidimensional Energy Poverty Index (PMEPI) based on Alkire-Foster method. With the definition evolving, scholars are gradually recognizing the serious consequences of energy poverty. As a key factor affecting the overall well-being of a household, energy poverty is likely to result in low indoor temperature, indoor air pollution and inadequate lighting, leading to health risks, lack of educational opportunities, and even development dilemmas for residents [14,15].

For many years, scholars have been exploring the negative impact of energy poverty on the health condition of residents. Healy analyzed the key factors contributing to mortality in winter, finding a strong correlation between energy poverty and increased mortality [16]. Further, Liddell found that people in energy poverty were more likely to suffer from respiratory diseases such as influenza, bronchitis, and asthma [17,18]. Scholars have reached a consensus that the main consequence of energy poverty are health risks.

In addition to the health risks, residents in energy poverty have to spend more time and opportunity costs collecting primitive solid fuel due to the lack of modern energy service [19], thus leading to a decrease of time allocated to learning and productive activities [20,21]. Many studies suggested that learning behavior was negatively associated with energy poverty. Khandker has found that residents without electricity supply had less learning time and completed schooling years [22]. For children, energy poverty even causes a decline in their academic performance [23].

However, the negative impacts caused by energy poverty are far more than health condition or learning behavior. In modern society, almost all domestic activities require energy supply. In the case of energy shortage, lighting, cleaning, laundry, communication, and other activities are all seriously impacted [24]. According to the ecological system theory of human development, the individual development of residents is the result of a complex set of contexts, indicating that the household environment shaped by energy poverty is likely to have a significant impact on individual development [25,26]. Accordingly, Acheampong has investigated the association between energy accessibility (one dimension of energy poverty) and human development index among 79 countries, and found that modern energy service such as electricity can effectively contribute to human development [27]. Nevertheless, such macro studies at country level can hardly reveal how energy poverty is associated with individual development. Further research is needed to fill the current gap.

Only with a comprehensive understanding of the consequences resulting from energy poverty can governments implement targeted policies. Beyond the direct effect of energy poverty on individual development, health condition and learning behavior may also mediate this relationship considering the existing research basis. Therefore, we have introduced health condition and learning behavior as mediating variables to analysis the specific mechanisms. In summary, this paper uses the data obtained from Chinese General Social Survey (CGSS) to conduct quantitative research, trying to explore the answers to following questions: (1) Does energy poverty have a negative impact on the individual development of the residents? (2) If so, what are the specific mechanisms in the impact of energy poverty on individual development?

The contributions of this paper are shown as follows: Firstly, most scholars have focused on the health risks caused by energy poverty, but paid little attention to the impact on individual development [28]. This paper hopes to enrich existing studies, and systematically explore how energy poverty influences individual development. Secondly, compared with Acheampong’s research, the inclusion of two mediating variables, health condition and learning behavior, enables this paper to conduct an in-depth mechanism analysis [27]. Last but not least, based on individual-level data, this paper can provide more reliable evidence than previous country-level studies [27]. Overall, this paper aims to shed light on the negative impacts of energy poverty on individual development, and provide some insights for governments to understand energy poverty.

## 2. Theoretical Bases and Hypothesis

Energy ladder model and need hierarchy theory are the theory bases of this research. As a classical theory to study the dynamics of fuel switching, energy ladder model has been widely discussed since the 20th century [29]. It divided energy into three types (primitive type, transition type and advanced type), and pointed out that there were a close correlation between energy type used in daily life and residents’ socio-economic status [30]. Need hierarchy theory was proposed by Maslow. He argued that the need for an individual could be arranged in a hierarchy. Only after the lower level needs are met, would individuals have the motivation to pursue the higher level needs [31,32]. Energy and individual development belong to different levels of need, and it is obvious that the satisfaction of energy needs may facilitate the residents’ pursuit for individual development.

### 2.1. Relationship between Energy Poverty and Individual Development

Previous literature noted that energy poverty was likely to have a negative impact on individual development. According to Chang’s study, energy-poverty families are characterized by high energy expense and low energy efficiency, and these dilemmas has shown a strong self-reinforcing tendency [33]. High energy expense will take up the spending that should be devoted to residents’ development activities, such as purchase of books or part-time education, making it difficult for the residents to improve their social-economic status [34]. As the United Nations stated in a report, energy poverty restricts one’s capabilities to realize his/her full potential [35,36]. On the other hand, energy poverty is the primary source of cumulative stress and negative emotion [37]. Zhang’s study found that energy poverty could negatively affect the class identity and fairness perception of the residents [38]. These pessimistic perceptions will make residents lack the intrinsic motivation to implement productive activities and improve their socioeconomic status. Therefore, the negative impact of energy poverty on individual development can be explained by both internal and external aspects. In addition, the energy ladder model states that one’s development can actually be seen as a process of climbing energy ladder to the higher level, supporting the above points to a certain extent [30].

### 2.2. Mediating Effect of Health Condition

The causality between energy poverty and health condition has been widely explored [37]. It has been proved that inadequate heating in energy-poverty families is significantly negatively associated with health condition [39]. By analyzing mortality data from the United Kingdom, Wilkinson has found that residents living in inadequately heated houses are more likely to die from cardiovascular disease [40]. For another, energy poverty also impairs residents’ health condition by polluting indoor air. In many cases, energy poverty means that a family can only use solid fuels for heating and cooking, leaving residents vulnerable to many diseases [41]. Peabody has conducted a large cross-sectional study of rural China, and found that indoor combustion of coal was associated with poorer health condition [42]. In fact, there are 38 million disability-adjusted life years lost per year around the world because of solid-fuel indoor air pollution [43]. Correspondingly, people with poorer health condition have to spend more money on medical care, which further reduces their ability to improve their living conditions and enhance their social-economic status [36,38]. Therefore, health condition is thus considered to mediate the relationship between energy poverty and individual development.

### 2.3. Mediating Effect of Learning Behavior

Energy poverty causes residents to spend a lot of time on fuel collection, which makes them allocate little time for regular learning. Adequate nighttime lighting is crucial to learning. However, energy poverty may hinder the learning behavior of the residents by reducing nighttime lighting [44,45]. On the other hand, a suitable environment is extremely important for the formation of learning behavior [46]. According to the need hierarchy theory, individuals must satisfy lower-level needs of themselves before pursuing the higher-level needs [47]. It is obvious that residents have no motivation to engage in creative activities such as learning new knowledge in a cold and damp environment caused by energy poverty. Further, numerous studies have confirmed that learning behavior is closely related to individual development [48,49]. Based on above analysis, we argue that learning behavior may also mediate the relationship between energy poverty and individual development.

### 2.4. Serial Mediating Effect of Learning Behavior and Health Condition

Learning behavior has been proven to have positive impacts on health condition. According to general theories of agency and self-efficacy, learning can mildly change people’s ingrained attitudes and behaviors to some extent, and thus improve their health [50]. Scholars have observed significant improvements in health condition among the people who engage in learning [51]. Aldridge conducted a survey of active adult learners, and found that about 90 percent of the respondents had experienced health benefits from learning [52]. Hence, we hypothesize that learning behavior and health condition can serially mediate the relationship between energy poverty and individual development.

### 2.5. Hypothesis of Current study

This study aims to explore the impact of energy poverty on individual development and the specific mechanisms of the relationship. Based on the theoretical analysis and previous studies, we propose the following hypotheses.

**Hypothesis** **1 (H1).** *Energy poverty has a negative effect on individual development.*

**Hypothesis** **2 (H2).** *Learning behavior mediates the relationship between energy poverty and individual development.*

**Hypothesis** **3 (H3).** *Health condition mediates the relationship between energy poverty and individual development.*

**Hypothesis** **4 (H4).** *Learning behavior and health condition serially mediates the relationship between energy poverty and individual development.*

## 3. Methodology

### 3.1. Serial Mediation Model

**Figure 1:**
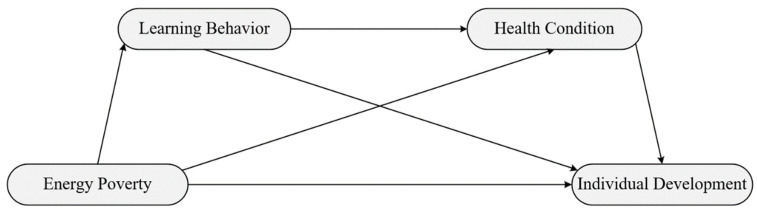
Serial mediation model.

As shown in Figure 1, we have developed a serial mediation model with two mediators to verify above hypotheses [53]. Learning behavior and health condition are defined as the first and second mediators separately. According to the serial mediation model and corresponding quantification method, we can figure out whether and how energy poverty affects individual development.

### 3.2. Data Source

Data used in this study are derived from the Chinese General Social Survey (CGSS). CGSS is an authoritative survey project conducted by National Survey Research Center at Renmin University of China. In compliance with multistage stratified sampling, CGSS has been continuously collecting cross-sectional data from different provinces [54,55]. In addition to the core modules, CGSS conducted in different years contains different modules to help scholars and governments investigate emerging social issues in each period. Specially, energy module was included in the CGSS2015, providing a data foundation for energy poverty study of China. We obtained the CGSS 2015 dataset containing 10,968 samples from the official website (http://cgss.ruc.edu.cn/ accessed on 15 July 2021). Specifically, the surveyors randomly selected one-third of the 10,968 respondents to ask questions about energy consumption. Some of the selected samples did not contain all of the key variables that this study needs. Therefore, we dropped 8679 samples, which did not ask related questions or did not contain key variables, and obtained a final 2289 valid samples. Although a certain number of samples were dropped, 2289 samples retained in this study were adequate enough to support the following quantitative analysis. According to CGSS2015 dataset, we can get a comprehensive understanding of the energy consumption situation in a given individual’s household, thus effectively determine whether the individual’s household is in energy poverty or not. Therefore, we are able to explore the impact of energy poverty on the individual development of residents by following quantitative methods.

### 3.3. Variable Measures

#### 3.3.1. Individual Development (Dependent Variable)

The individual development variable was obtained with the question “Compared to three years ago, how do you think your socioeconomic status has changed?” The respondents’ answers ranged from “has risen”, “has not changed” to “has declined”. This question has been frequently used in previous studies based on CGSS data [56]. To facilitate the data analysis, we recoded “has risen” as 3, “has not changed” as 2, and “has declined” as 1. The higher score implied that the individual development of the residents was better.

#### 3.3.2. Energy Poverty (Independent Variable)

Energy poverty should include two equally important dimensions consisting of accessibility and affordability [57]. Accessibility reflects the residents’ dependence on solid fuels, while affordability reflects the residents’ difficulty in paying for necessary energy [27,58]. Based on previous studies, we treated residents using solid fuels for cooking or spending more than 10% of their family income on energy as being in energy poverty [57]. More specifically, the level of energy poverty was classified as “not in energy poverty” = 1, “in mild energy poverty (in one of the two energy poverty types)” = 2 and “in severe energy poverty (in both types of energy poverty)” = 3.

#### 3.3.3. Learning Behavior and Health Condition (Moderating Variables)

The learning behavior variable was obtained from the question set “In the past year, did you often do the following in your free time?” One sub-question of this question set was the frequency of “learning”. Respondents were asked to answer this sub-question with a 5-point Likert scale ranging from “never” (=1) to “frequently” (=5). The higher score implied that the residents spent more time on learning.

The health condition variable was obtained from the question “How do you feel about your current health condition?”. Respondents were also asked to answer the question with a 5-point Likert scale ranging from “very unhealthy” (=1) to “very healthy” (=5). Accordingly, higher score implied better health condition of the residents.

#### 3.3.4. Sociodemographic Characteristics (Covariates)

Based on previous studies on energy poverty and individual development [36,59,60,61], we selected six sociodemographic characteristics including age, gender (male = 0, female = 1), educational level (primary school and below = 1, middle school = 2, high school = 3, junior college = 4, bachelor degree and above = 5), marital status (not having a spouse = 0, having a spouse = 1), family income and region (urban area = 0, rural area = 1) as covariates.

### 3.4. Data Analysis

Descriptive statistics including percentage, mean and standard deviation of the variables was shown in the following section. Meanwhile, we used Pearson’s correlation coefficients matrix to examine the correlation among key variables. The descriptive statistics and correlation analysis were conducted by IBM SPSS Statistics 26.0 (SPSS, IBM Corp, Armonk, NY, USA). Further, PROCESS macro version 3.5 was used to conduct serial mediating effects analysis [62]. Based on model 6 of PROCESS, we can examine the direct/indirect effects of energy poverty on individual development via mediating variables of learning behavior and health condition [63]. The bootstrap method (95% confidence intervals, 5000 bootstrap samples) was conducted to estimate the coefficients of indirect effects. Specifically, the indirect effect was statistically significant if 0 was not within the 95% CI.

## 4. Results

### 4.1. Descriptive Statistics and Correlation Analysis

The descriptive statistics is presented in Table 1. After sample screening and eliminating, a total of 2289 valid samples were included in the study. In total, 35.08% of the respondents achieved a good individual development. More than half of the respondents were in mild energy poverty (40.15%) or severe energy poverty (10.62%). The average score of learning behavior was 1.90, indicating that spontaneous learning is still not the main activity in residents’ spare time. The average score of health condition was 3.59. In terms of sociodemographic characteristics, the proportion of male respondents (47.27%) was similar to that of female respondents (52.73%). The average age of the respondents was 57 years old, and the average family annual income was RMB 66,932. In total, 81.61% of the respondents have a spouse. In total, 39.49% of the respondents had an educational level of primary school and below, while only 6.12% of the respondents had an educational level of bachelor degree and above. In addition, 53.21% of the respondents lived in an urban area, and 46.79% in a rural area.

The correlation analysis among variables is presented in Table 2. Energy poverty is significantly negatively correlated with individual development (r = −0.053, *p* = 0.05), learning behavior (r = −0.250, *p* = 0.01) and health condition (r = −0.201, *p* = 0.01). Learning behavior shows significant positive correlations with individual development (r = 0.080, *p* = 0.01) and health condition (r = 0. 236, *p* = 0.01). Health condition shows a significant positive correlation with individual development (r = 0. 123, *p* = 0.01). Correlation analysis provided a basic support for the following hypothesis validations.

### 4.2. Serial Mediating Effects Analysis

**Figure 2:**
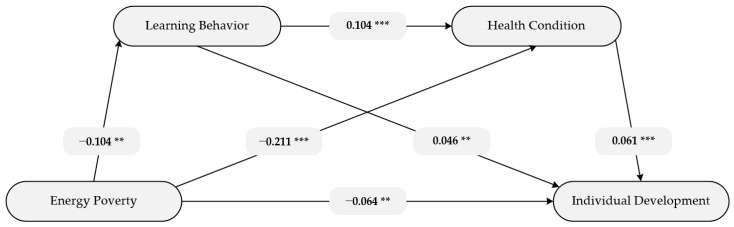
Overall pathways and coefficient. Note. ** *p* < 0.01, *** *p* < 0.001.

Serial mediating effects analysis was conducted to verify the mediating effects of the two mediating variables, learning behavior and health condition. The results of serial mediating effects analysis are shown in Figure 2.

Figure 2 illustrates that energy poverty was significantly negatively correlated with learning behavior (r = −0.104, *p* < 0.01), health condition (r = −0.211, *p* < 0.001) and individual development (r = −0.064, *p* < 0.01). Learning behavior positively affects health condition (r = 0.104, *p* < 0.001) and individual development (r = 0.046, *p* < 0.01). Health condition positively affects individual development (r = 0.061, *p* < 0.001).

The direct and indirect effects of energy poverty on individual development are presented in Table 3. Of all the results, 0 is not within the 95% CI, indicating that estimated effects are significant. The direct effect of energy poverty on individual development is −0.0641 (CI: −0.1084, −0.0199). Hypothesis 1 is confirmed. In terms of indirect effect, learning behavior has a significant mediating effect on the relationship (indirect effect = −0.0047, 95% CI: −0.0091, −0.0012), while health condition also has a significant mediating effect (indirect effect = −0.0128, 95% CI: −0.0209, −0.0060). Thus, Hypothesis 2 and Hypothesis 3 are confirmed. In addition, there is a significant indirect effect of energy poverty on the individual development via the serial mediation of learning behavior and health condition (indirect effect = −0.0007, 95% CI: −0.0013, −0.0002), supporting Hypothesis 4. In summary, the hypotheses presented above are all confirmed. In other words, learning behavior and health condition can partially mediate the relationship between energy poverty and individual development.

**Figure 3:**
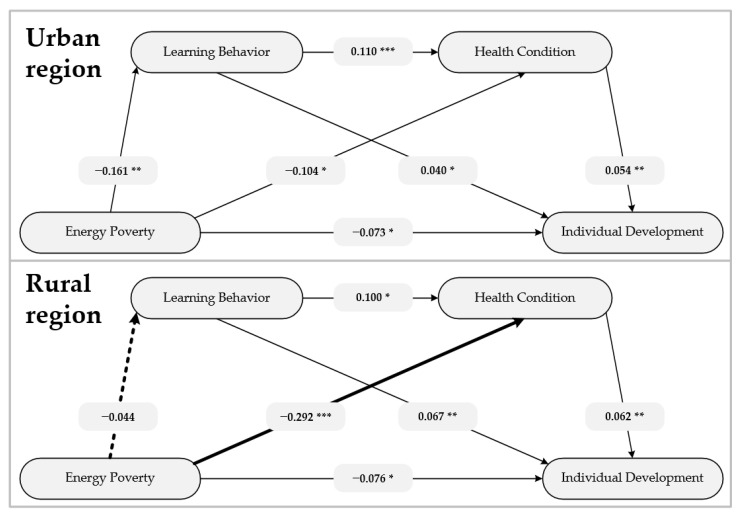
Pathways and coefficient in urban and rural regions. Note. * *p* < 0.05, ** *p* < 0.01, *** *p* < 0.001.

To analyze whether sociodemographic feature influences the relationship between energy poverty and individual development, we have conducted pathways analysis for urban and rural region respectively. The results are shown in Figure 3. Energy poverty significantly directly affects individual development in both urban and rural regions. However, in terms of indirect effects, the results in urban and rural regions have shown notable differences. For urban regions, the indirect effects are consistent with the initial hypotheses that learning behavior and health condition can independently or serially mediate the relationship between energy poverty and individual development. For rural region, the mediating effect of learning behavior is not significant, while the mediating effect of health condition is quite strong.

## 5. Discussion

Energy plays an irreplaceable role in modern society. As a new type of poverty, energy poverty has negative impacts on the residents’ life from various aspects. A comprehensive understanding of these impacts is the top priority in energy poverty governance. This study has explored the impact of energy poverty on individual development and the specific mechanisms using a serial mediation model. Large-scale sample data obtained from Chinese General Social Survey guaranteed the reliability of this study.

This study has broadened the previous literatures on both research content and methodology. There have been many quantitative studies focusing on the impacts of energy poverty on health condition [8,57], but studies about the correlation among energy poverty, learning behavior, and individual development have been limited to the qualitative perspective [64]. This study has developed an integrative framework about energy poverty’s negative impacts, and is the first to explore the relationship between energy poverty and individual development from a quantitative perspective. On the other hand, previous studies on energy poverty usually utilized relatively simple models that only directly examined the relationship between energy poverty and a specific dependent variable, which inevitably resulted in variable omission and estimation errors. The use of the serial mediation model in this study enables us to comprehensively incorporate the various impacts of energy poverty, and obtain more reliable estimate results [65,66].

It is found that energy poverty has a significant negative correlation with individual development, and learning behavior and health condition can independently or serially mediate the correlation. Specifically, several meaningful conclusions can be drawn as follows.

Firstly, the direct effect of energy poverty on individual development indicates that energy poverty can strongly and negatively affect one’s development. Considering that the samples used in this study actually cover all age groups of the residents, we argue that the impact of energy poverty on individual development is experienced almost throughout someone’s lifetime. This finding is a further expansion of previous research that mainly focused on the development of children and women [23,67]. Due to the inclusion of income, region and other covariates, this study also confirms that, even with similar income, residents’ pursuit and utilization of modern fuel can drive up their socioeconomic status. Therefore, this study has enriched the energy ladder theory and provided new, fact-based evidence to the discussion of the link between energy consumption and socioeconomic status. With regard to the governance of energy poverty, the government should not view energy poverty as a short-term predicament of a person or a family, but treat energy poverty as an obstacle factor for long-term development of residents, and take proactive measures to prevent them from falling into the “vicious cycle” of energy poverty.

Secondly, learning behavior and health condition can independently or serially mediate the relationship between energy poverty and individual development. Health condition has the strongest mediating effect. Previous studies showed that energy poverty caused negative effects on residents’ health through polluted indoor air and low indoor temperature [68,69]. Meanwhile, studies of Liu and Campino confirmed that health condition was crucial for individual development [70,71]. This study has revalidated and integrated previous findings, and emphasized that energy inequalities are likely to lead to health inequalities and development inequalities. Compared to health condition, the coefficient between energy poverty and learning behavior is smaller, that is, the mediating effect of learning behavior is weaker. By econometric methods, Zhang has demonstrated that energy poverty can affect children’s academic performance [23]. This study has expanded the concepts and scopes of Zhang’s study, broadening academic performance into learning behavior, from children into the adult population. The mediating effect of learning behavior has confirmed and enriched Maslow’s need hierarchy theory: Energy is the basic need of the residents in modern society, and only when energy need is satisfied, residents can pursue higher-level needs such as learning and self-actualization. Some interesting results are obtained in the pathways analysis for rural and urban regions. For rural region, learning behavior cannot mediate the relationship between energy poverty and individual development, while health condition plays a stronger mediating role. Compared with urban regions, most rural regions are less developed [72]. Due to the universal lack of attention to learning and self-improvement, rural residents’ learning behaviors are clearly less frequent than that of urban residents, thus weakening the mediating effect of learning behavior [73]. On the other hand, while urban energy-poverty residents may use coal as a cooking/heating fuel, energy-poverty residents in rural regions do not even have access to coal and have to collect straw, firewood, and animal dung as energy sources [74,75]. These extremely rudimentary fuels have undoubtedly caused greater health risks, and thus make the mediating effect of health condition stronger.

The findings of this study have provided some policy implications about energy poverty governance: Energy poverty encompasses two dimensions including accessibility and affordability. Energy poverty does not only occur in rural areas with poor infrastructure, but also occurring in urban low-income households. Therefore, to help residents achieve better development, the government should improve energy infrastructure in rural areas while alleviating the energy price burden of the residents as much as possible. Furthermore, governments should not only be fully aware of various impacts of energy poverty, but also set priorities for coping with these impacts. In consideration of the strong impact of energy poverty on health condition in rural regions, governments should first and foremost provide necessary supports for the residents with poor health condition due to energy poverty, followed by persuading residents to use modern energy to facilitate their own learning activities.

Nevertheless, there are several research limitations that should be improved in the future. First, due to the questionnaire limitations of CGSS, this study used a self-reported indicator to represent individual development, which inevitably leads to subjective bias. Scholars are suggested to use composite objective indicators to improve the reliability of research in the future. Second, in addition to health condition and learning behavior, there are also some psychological factors such as subjective well-being that could be correlated with energy poverty [58]. It is recommended that future studies integrate psychological factors into their research framework to ensure comprehensiveness. Third, we recommend that future studies further explore the limitation of educational opportunities caused by energy poverty according to a well-designed questionnaire survey to clarify the relationship between them. Last but not least, compared with panel data, we cannot thoroughly examine the causal relationships among variables by cross-sectional data used in this study [26]. Future studies should consider including multi-year panel data when the data source is reliable.

## 6. Conclusions

This study has explored the impact of energy poverty on individual development and the specific mechanisms of this process. Drawing upon energy ladder model and need hierarchy theory, we found that energy poverty can negatively impact individual development. Further, learning behavior and health condition were found to be correlated with energy poverty. Both of these factors can independently or serially mediate the relationship between energy poverty and individual development. Health condition has the stronger mediating effect, whereas the mediating effect of learning behavior is weaker. Moreover, the health condition of rural residents is more vulnerable to energy poverty due to the shortage of modern fuels. This study may contribute to a better understanding of the consequences of energy poverty in government and academia.

## Figures and Tables

**Table 1:** Descriptive statistics.

Variable	Categories		Percentage	Mean	Standard Deviation
Individual development	Socioeconomic status has declined	(1)	10.18	2.25	0.63
	Socioeconomic status has not changed	(2)	54.74		
	Socioeconomic status has risen	(3)	35.08		
Energy poverty	Not in energy poverty	(1)	49.24	1.61	0.67
	In mild energy poverty	(2)	40.15		
	In severe energy poverty	(3)	10.62		
Learning behavior	Never	(1)	46.61	1.90	1.05
	Rarely	(2)	28.27		
	Sometimes	(3)	15.47		
	Often	(4)	7.60		
	Frequently	(5)	2.05		
Health condition	Very unhealthy	(1)	2.97	3.59	1.08
	Unhealthy	(2)	15.81		
	Normal	(3)	21.97		
	Healthy	(4)	37.96		
	Very healthy	(5)	21.28		
Gender	Male	(0)	47.27	0.53	0.50
	Female	(1)	52.73		
Educational level	Primary school and below	(1)	39.49	2.11	1.18
	Middle school	(2)	29.84		
	High school	(3)	17.39		
	Junior college	(4)	7.16		
	Bachelor degree and above	(5)	6.12		
Marital status	Not having a spouse	(0)	18.39	0.82	0.39
	Having a spouse	(1)	81.61		
Region	Urban area	(0)	53.21	0.47	0.50
	Rural area	(1)	46.79		
Age				56.90	16.09
Income				66,932.08	250,613.51

**Table 2:** Correlation analysis.

Variables	Individual Development	Energy Poverty	Learning Behavior	Health Condition
Individual development	1			
Energy poverty	−0.053 *	1		
Learning behavior	0.080 **	−0.250 **	1	
Health condition	0.123 **	−0.201 **	0.236 **	1

Note. * *p* < 0.05, ** *p* < 0.01.

**Table 3:** The serial mediating effects analysis among variables.

Pathway	Effect	SE	95%CI	
			LLCI	ULCI
Direct effect	−0.0641	0.0226	−0.1084	−0.0199
Indirect effect				
EP → Learning behavior → Individual development	−0.0047	0.0020	−0.0091	−0.0012
EP → Health condition → Individual development	−0.0128	0.0038	−0.0209	−0.0060
EP → Learning behavior → Health condition → Individual development	−0.0007	0.0003	−0.0013	−0.0002

Note. EP means energy poverty, SE means standard error, LLCI means lower limit confidence interval, ULCI means upper limit confidence interval.

## Data Availability

Not applicable.
